# Tiny Subsamples and Upsampling Tame Big Data Evolutionary Analysis in Phylogenomics

**DOI:** 10.64898/2026.06.21.733599

**Published:** 2026-06-22

**Authors:** Sudhir Kumar, Koichiro Tamura, Sudip Sharma

**Affiliations:** 1 Institute for Genomics and Evolutionary Medicine, Temple University, Philadelphia, PA 19122, USA; 2 Department of Biology, Temple University, Philadelphia, PA 19122, USA; 3 Department of Biological Sciences, Tokyo Metropolitan University, Tokyo, Japan; 4 Research Center for Genomics and Bioinformatics, Tokyo Metropolitan University, Tokyo, Japan

**Keywords:** phylogenomics, confidence limits, model selection, parameter estimation, hypothesis testing, incomplete lineage sorting

## Abstract

Long runtime, high memory demands, and reliance on high-performance computing increasingly limit the evolutionary analysis of long phylogenomic datasets. We review a scalable framework based on phylogenomic subsampling and upsampling (PSU), in which many small subsamples of sites from a long concatenated sequence alignment are extended by upsampling prior to inference, and the resulting analyses are then aggregated to obtain stable evolutionary estimates. PSU exploits a useful distinction between the computational burden and the inferential power of statistical methods in molecular phylogenetics: computational cost is strongly influenced by the number of distinct site patterns in the concatenated alignment, whereas statistical power depends primarily on the amount of evolutionary information represented by sites and substitutions. By reducing the former while restoring the latter through upsampling, PSU can approximate many full-data analyses at substantially lower computational cost. Evidence from simulated and empirical datasets shows that PSU can accurately estimate bootstrap support values, select optimal substitution models, test evolutionary hypotheses, and infer branch lengths, divergence times, and associated uncertainty measures, while often reducing runtime and memory requirements by orders of magnitude. The same subsampling–upsampling–aggregation principle underlies all of these applications. PSU also provides distributions of inferred clade support across independent subsamples, enabling detection of concordant and conflicting phylogenetic signals that may remain hidden in conventional concatenated phylogenomic analyses. Adaptive procedures for selecting the subsample size, the number of subsamples, and the number of upsampling replicates make the framework practical across diverse datasets. We suggest that PSU is a general strategy for scalable phylogenomic inference across a broad range of statistical methods. By enabling rigorous analyses of genome-scale alignments on standard computing hardware, PSU expands access to computationally intensive evolutionary methods while reducing the environmental and infrastructural costs of big-data phylogenomics.

## Introduction

**Figure 1.**
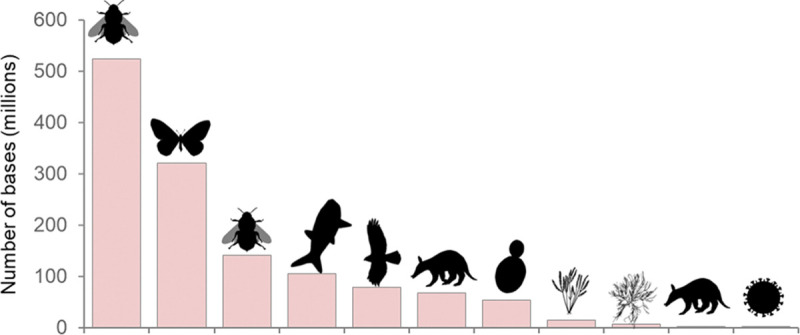
Phylogenomic datasets across the Tree of Life. Each bar corresponds to a phylogenomic dataset, with the bar height proportional to the number of bases (sites × sequences). Dataset references are in Supplementary Table S1.

Advances in sequencing technology and the rapid growth of sequence databases have made it increasingly easier to assemble large collections of sequences spanning thousands of loci across diverse organisms, individuals, and strains (Fig. 1). This expansion has transformed molecular phylogenetics into phylogenomics, which involves analyzing genome-scale alignments to infer evolutionary relationships, estimate divergence times, and investigate patterns of molecular evolution (Philippe et al. 2005; Kumar et al. 2012).

**Figure 2.**
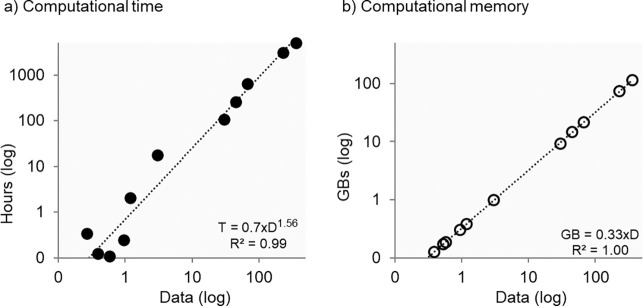
Increasing Computational demands. (**a**) Runtime and (**b**) memory requirements for ML analyses of empirical and simulated datasets. Data size (D) on the x-axis is the product of the number of sequences and the number of unique site patterns, as identical sites are collapsed during ML analysis. Source data are from Table 1 of Sharma and Kumar (2022).

The success of phylogenomics rests on a simple principle: longer alignments contain more evolutionary information and generally provide greater statistical power for estimating phylogenies and evolutionary parameters. However, the computational demands of analyzing these datasets increase dramatically with alignment size. This burden is particularly acute for computationally intensive methods such as maximum likelihood (ML; Fig. 2) and other sophisticated approaches. For example, selecting the best-fit nucleotide substitution model for an alignment of 394 kilobase pairs from 200 bird species (hereafter, the 200×394 dataset) required more than 10 days of computation (258 hours) and 14 GB of memory (Prum et al. 2015; Sharma and Kumar 2022). Similar computational demands are experienced when ML and other sophisticated methods are used to infer phylogenies, evaluate bootstrap support, and estimate divergence times (Sharma and Kumar 2021; Kumar 2022; Sharma and Kumar 2022). As phylogenomic datasets continue to grow, many analyses become impractical on standard desktop computers and increasingly depend on specialized high-performance computing resources.

These computational demands hinder discovery and reduce scientific rigor. Long runtimes discourage robustness checks across alternative models, assumptions, and data treatments, while reliance on specialized high-performance computing infrastructure limits accessibility for many researchers. Reproducibility also suffers when independent reanalyses require days of computation or more memory resources unavailable on standard desktops. Furthermore, repeated large-scale analyses carry environmental costs because computational time translates into energy consumption and carbon emissions (Kumar 2022). Computational innovations are needed to make evolutionary analysis scalable for big-data phylogenomics.

A key observation that has motivated recent advances in scalable phylogenomics is that computational burden and inferential power are often governed by distinct properties of a concatenated phylogenomic alignment. Computational cost depends strongly on the number of distinct site configurations across sequences (site patterns) that must be evaluated, whereas statistical power depends primarily on the total amount of evolutionary information represented by sites and substitutions. This distinction creates an opportunity: if one can preserve much of the statistical information contained in a large alignment while substantially reducing the number of distinct site patterns analyzed, it may be possible to obtain accurate evolutionary inferences at a fraction of the computational cost.

Phylogenomic subsampling and upsampling (PSU) is an emerging framework that exploits this separation. In PSU, many random subsamples of alignment sites are drawn from the full concatenated alignment without regard to gene boundaries, partitions, or prior biological assumptions. Each subsample is then expanded via upsampling prior to inference. This upsampling restores the full-alignment scale in terms of the total number of sites and substitutions represented, while retaining only a small fraction of the distinct site patterns found in the original dataset. Results from multiple subsamples are then aggregated to produce stable estimates. Because each analysis evaluates only a small subset of site patterns, computational requirements can be dramatically reduced without sacrificing statistical power. Traditional phylogenomic subsampling approaches generally analyze only the selected subset of sites. Because these reduced datasets contain fewer sites and substitutions than the full alignment, they often suffer a loss of statistical power and may require additional assumptions to relate their estimates and variances to those obtained from the full dataset (Sharma and Kumar 2021; Sharma and Kumar 2022). For these reasons, phylogenomic subsamples are frequently used to evaluate the stability of evolutionary inferences from concatenated datasets and to explore heterogeneity in phylogenetic signal across partitions and loci (Seo 2008; Faircloth et al. 2012; Song et al. 2012; Edwards 2016; Mongiardino Koch 2021; Lozano-Fernandez 2022).

PSU builds on the statistical foundation of the bag-of-little-bootstraps framework (Kleiner et al. 2014) but extends it to the distinct needs of phylogenomics. Initially adapted for estimating bootstrap confidence limits on genome-scale phylogenies (Sharma and Kumar 2021), PSU has subsequently been applied to substitution model selection and the detection of conflicting phylogenetic signals (Sharma and Kumar 2021; Sharma and Kumar 2025). Here, we demonstrate that it can be applied to test evolutionary hypotheses and to estimate branch lengths, divergence times, and their variances. These developments suggest that PSU is more than a bootstrap acceleration technique; it represents a general framework for scalable phylogenomic inference.

Like many widely used strategies in phylogenetics, PSU should be viewed as a computational approximation rather than an exact formulation of full-data inference. This places it alongside heuristic tree-search methods, approximate likelihood calculations, and numerical optimization procedures that routinely trade computational efficiency for practical scalability, e.g., (Strimmer and Von Haeseler 1996; Price et al. 2010a; Stamatakis 2014; Minh et al. 2020; Azouri et al. 2021; Kumar et al. 2023; Kumar et al. 2024). Its value, therefore, rests on empirical performance, convergence behavior, and practical utility across realistic datasets. In the sections that follow, we review the logic, applications, performance, and limitations of PSU as a unifying framework for scalable phylogenomic inference.

## Estimating bootstrap support using PSU

Estimating confidence in inferred evolutionary relationships was the first major application of the PSU framework. Bootstrap support value estimation is particularly attractive because it requires repeated analyses of datasets that are nearly as large as the original alignment, making computational demands grow rapidly with dataset size. PSU addresses this challenge by replacing full-alignment bootstrap analyses with analyses of many small site subsamples that are subsequently upsampled and aggregated. This application illustrates the general PSU strategy: construct computationally efficient representations of a large alignment, restore statistical scale through upsampling, and combine results across subsamples to obtain evolutionary estimates.

### Felsenstein’s bootstrap approach

**Figure 3.**
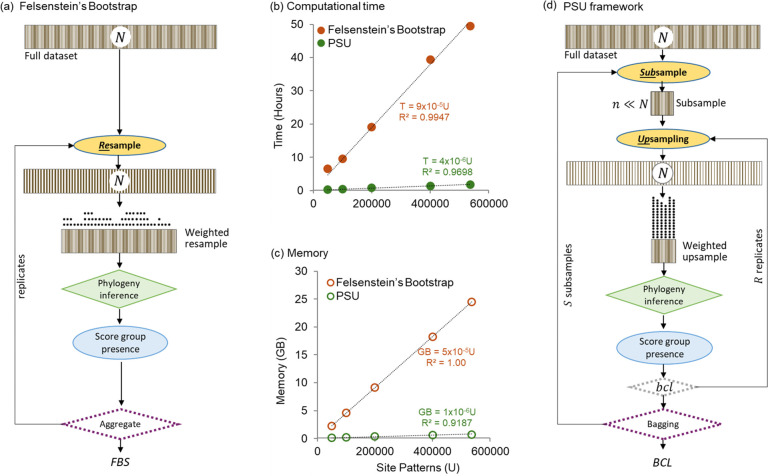
Flowcharts and computational demands of Bootstrap analysis. (**a**) In Felsenstein’s bootstrap, replicate datasets are generated by resampling sites from the full dataset (*N* sites), maintaining both dataset size and the site pattern diversity. Felsenstein’s bootstrap support (*FBS*) for a clade is the proportion of replicate phylogenies containing the clade of interest. (**b**) runtime and (**c**) memory usage for inferring ML phylogeny for one bootstrap replicate for datasets containing increasing numbers of unique site patterns (U) in the full alignment. (**d**) In the PSU framework, small subsamples of randomly selected *n* sites (*n* << *N*) are upsampled to full length (*N* sites). Clade support is estimated for each subsample (*bcl*), then aggregated across *S* subsamples to obtain the median of the *bcl* values (*BCL*), which is used to estimate *FBS*. Panels *b* and *c* show the time and memory required to analyze a PSU replicate dataset. Results are from the analysis of simulated datasets from a previous study (Tamura et al. 2012) containing 446 sequences and alignments ranging from 50,000 to 536,534 bases, as reported in Fig. 1b of Sharma and Kumar (2021).

Felsenstein (1985) adapted Efron’s (1979) bootstrap method to assess confidence in clades derived from molecular phylogenetic analysis. In Felsenstein’s bootstrap, R replicate alignments are generated by resampling sites with replacement from the full dataset of *N* sites. Each replicate dataset also has *N* sites. A phylogeny is inferred from each replicate dataset, and the proportion of replicates that reconstruct a particular clade is used as its bootstrap support (*FBS*) (Fig. 3a). This analysis evaluates the statistical stability of inferred clades under sampling variation introduced by resampling sites from the full dataset. Felsenstein’s bootstrap procedure is computationally expensive because hundreds of phylogenetic analyses must be conducted on bootstrap replicate datasets, each containing ~63.2% of the original sites. This is because the probability that a site is not chosen in a given draw is (1 – 1/*N*), and thus the probability that it is never chosen in all *N* draws is (1 – 1/*N*)^*N*^. Therefore, the probability that the site appears at least once is 1 - (1 – 1/*N*)^*N*^, which approaches 1 - *e*^−1^ ≈ 0.632 as *N* becomes large. Thus, each bootstrap replicate contains about 63.2% of the original sites and, thus, site patterns.

Since computational time scales linearly with the number of sites in the dataset (Fig. 3b), Felsenstein’s bootstrap analysis with just 100 replicates is expected to take more than 63 times longer than a single ML phylogeny inferred from the full dataset. These requirements escalate with more replicates and can become onerous for very long phylogenomic alignments (Fig. 3b). Memory requirements per replicate also increase linearly with sequence length, which can far exceed the memory available on standard desktops (Fig. 3c). These demands motivated the need for alternative strategies that can generate accurate estimates of bootstrap support while greatly reducing computational costs.

### PSU-based bootstrap support estimation

**Figure 4.**
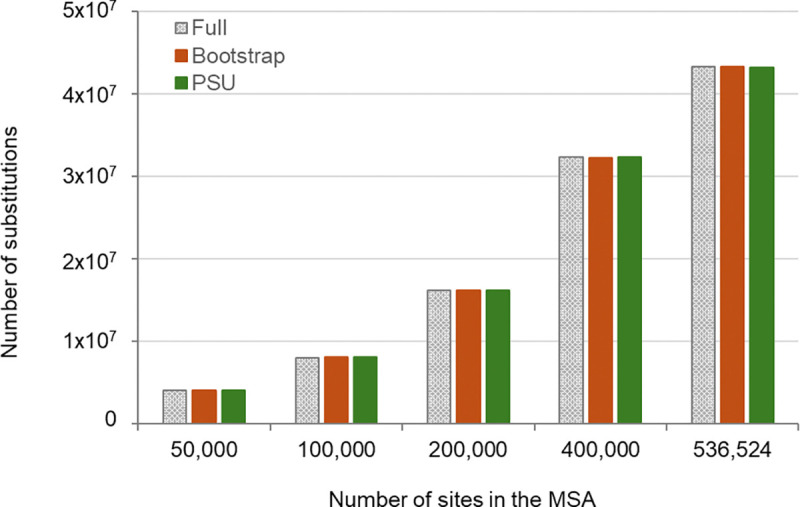
Number of substitutions in PSU datasets. The number of substitutions in PSU replicate datasets (green) as compared to those in the standard bootstrap replicate datasets (red) and the full dataset (grey). Results are from the analysis of simulated datasets containing 446 species and MSAs with 50,000 to 536,534 sites, as reported by Sharma and Kumar (2021). The number of substitutions is the sum of ML estimates of branch lengths multiplied by the number of sites.

Sharma and Kumar (2021) adapted and advanced the bag-of-little-bootstraps approach of Kleiner et al. (2014) for phylogenomic analysis (Fig. 3d). Each PSU analysis begins by selecting a random subset of sites (*n* << N) from the full alignment. Bootstrap replicate datasets are generated by resampling N sites with replacement from that subsample. Each upsampled replicate alignment therefore matches the full dataset length (*N*) while containing only n distinct sites present in the subsample. This upsampling step is central to the PSU framework because it restores the statistical scale of the analysis without the computational burden of the full alignment. Although only a fraction of the original site diversity is retained, the total number of sites and substitutions included in the analysis is restored to approximately full-data levels. As shown in Figure 4, PSU replicate datasets contain numbers of substitutions comparable to those found in conventional bootstrap replicates of the full dataset. Thus, PSU deliberately trades complete site diversity for computational efficiency while preserving much of the statistical scale needed for confidence estimation.

In the PSU analysis, multiple upsampled datasets are generated from each subsample. A phylogeny is inferred for each PSU replicate, and the proportion of PSU trees containing a specific clade yields the subsample bootstrap confidence level (*bcl*). Because each subsample includes only a fraction of the sites in the full dataset, multiple subsamples must be analyzed to obtain a stable estimate of full-data bootstrap support. Thus, the collection of bcl values across these subsamples is aggregated to estimate *FBS* for a given clade.

The way subsample *bcl* values are aggregated is important. Sharma and Kumar (2021) found that the mean *bcl*, although strongly correlated with *FBS*, tended to underestimate high *FBS* values and overestimate low *FBS* values. The median of the subsample *bcl* values, denoted *BCL*, provided a better approximation of conventional bootstrap support. This difference likely reflects the indicator nature of clade support: each replicate tree either contains a clade or does not, unlike the continuous estimators emphasized in the original bag-of-little-bootstraps framework of Kleiner et al. (2014). The Appendix provides a simple illustration of why median aggregation can outperform mean aggregation for such indicator-style confidence measures.

*BCL* estimation requires only a fraction of the time and memory needed for full-data bootstrap analysis. For the 200×394 dataset, PSU reduced computation time by 79% and memory use by 95% (Table 1), with similar gains reported for other empirical datasets (Sharma and Kumar 2021). In many cases, subsampling fewer than 5% of sites was sufficient, and approximately 10 subsamples, each with about 10 upsampling replicates, produced stable results. These reduced memory demands also make PSU analyses easier to parallelize across cores, further decreasing wall-clock time. PSU is therefore especially useful when full-data bootstrap analysis would exceed desktop memory limits or require days of computation.

### Distributions of clade support from PSU

**Figure 5.**
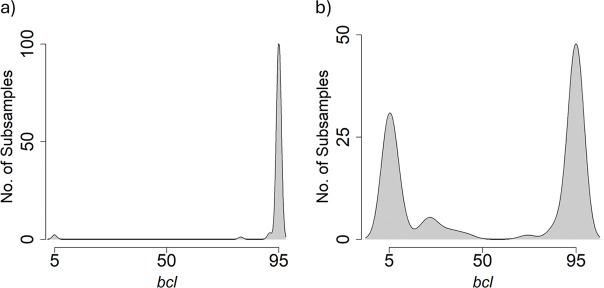
Distribution of subsample bootstrap support values (bcl) for two rodent clades. (**a**) Consistently high support across subsamples for a clade. (**b**) A distribution indicating conflicting phylogenetic signals across subsamples for the inferred clade. Results are from Sharma and Kumar (2025) and correspond to clades R1 and R1’ in the rodent phylogeny presented in Figure 3 in Sharma and Kumar (2025)

Unlike full-data bootstrapping, which reports a single support value for each clade (*FBS*), PSU produces a distribution of support values across subsamples for the selected subsample size (*n*). Some clades show consistently high support across subsamples, indicating strong genome-wide concordance (Fig. 5a). Others show broad or multimodal distributions, with some subsamples supporting the clade and others contradicting it (Fig. 5b). Such heterogeneity can reflect alignment or orthology errors, model misspecification, hidden biases, incomplete lineage sorting, or other sources of phylogenetic discordance (Lanfear and Hahn 2024; Sharma and Kumar 2024; Sharma and Kumar 2025). PSU detects this heterogeneity directly from random site subsamples rather than from predefined genes or partitions.

The distribution of *bcl* values can be used to estimate Net Bootstrap Support, *NBS*, a measure designed to reduce overconfidence in concatenated phylogenomic analyses (Sharma and Kumar 2025). Whereas *BCL* uses the median *bcl* to approximate conventional bootstrap support (*BCL* ~ *FBS*), NBS is calculated as the mean of the subsample *bcl* distribution. This distinction is important. The median is useful when the goal is to approximate *FBS*, but it can downplay conflicting subsamples. The mean incorporates the entire distribution, including subsamples that weakly support or contradict a clade, and, therefore, provides a theoretically supported measure that is more sensitive to phylogenetic conflict (Sharma and Kumar 2022).

**Figure 6.**
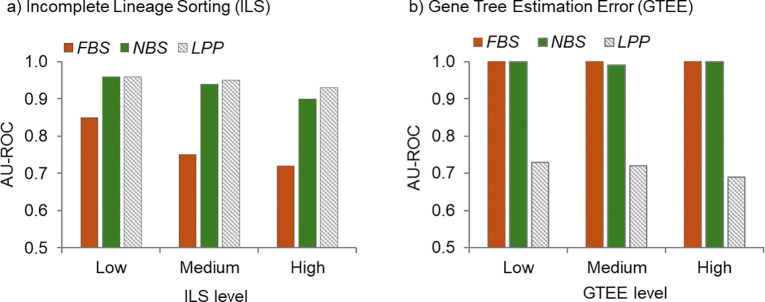
Accuracy of phylogeny support measures under heterogeneous conditions. Performance of support metrics in distinguishing correct and incorrect clades under varying levels of (**a**) incomplete lineage sorting (ILS) and (**b**) gene tree estimation error (GTEE), redrawn using data in Figures 7 and 8 of Sharma and Kumar (2025). Simulated datasets were obtained from Mirarab et al. (2014) and simulated with low, medium, and high levels of ILS using a species tree of 37 mammalian species within a multispecies coalescent framework. Ten datasets were analyzed from each ILS category, each comprising 100 genes (1,600 sites per gene). Simulated datasets with GTEE were obtained from Shen et al. (2021), in which branch lengths were reduced to introduce low, medium, and high GTEE levels, with 1,000 gene sequence alignments for each category. Net Bootstrap Support (*NBS*), derived from the mean of subsample support values, incorporates both supporting and conflicting signals and achieves performance comparable to or exceeding local posterior probability (*LPP*) produced by multispecies coalescent (MSC)-based approaches under challenging conditions.

Analyses of some simulated datasets suggest that NBS can perform similarly to MSC methods across phylogenomic datasets generated under low, moderate, and high levels of ILS (Fig. 6a) (Sharma and Kumar 2025). Interestingly, *NBS* could surpass multispecies coalescent (MSC)-based local posterior probabilities when gene tree estimation error (GTEE) was high (Fig. 6b). This outcome is biologically plausible because MSC methods depend on the quality of the input gene trees, which can become unreliable when individual gene alignments are short or contain few phylogenetically informative substitutions (Simmons and Gatesy 2015; Shen et al. 2021; Sharma and Kumar 2025). Because NBS does not depend directly on estimated gene trees, its performance is not affected by gene-tree estimation error in the same way as MSC-based approaches.

In addition to ILS and GTEE, one or a few loci with unusual phylogenetic histories, alignment issues, and hidden biases can drive bootstrap support for incorrect or contentious clades in concatenated analyses of hundreds of loci (Brown and Thomson 2016; Shen et al. 2017; Sharma and Kumar 2024; Sharma and Kumar 2025). In the PSU analysis, some subsamples will exclude such disruptive loci and sites, thereby reducing support for spurious clades driven by those sites (Sharma and Kumar 2025). In these cases, *NBS* is expected to be lower than *FBS*, as observed in the concatenated analysis of multiple empirical datasets (Sharma and Kumar 2025). Thus, *NBS* can serve as a practical way to reduce overconfidence while simultaneously revealing heterogeneity in phylogenetic signals across the alignment.

### Phylogenomics without data partitions

Conventional approaches for addressing phylogenetic heterogeneity often divide an alignment by gene, codon position, genomic region, or other biologically defined categories. These partitions may then be modeled separately in concatenated analyses or used to reconstruct gene trees, which are summarized using consensus or multispecies coalescent methods (Bull et al. 1993; Chippindale and Wiens 1994; Brandley et al. 2005; Gadagkar et al. 2005; Lanfear et al. 2017). Such strategies are powerful and widely used, but they also require decisions about how to partition the data, and different partitioning schemes can lead to different conclusions. In addition, fitting separate models to many partitions or estimating many gene trees can be computationally demanding, and short partitions may suffer from gene tree estimation error (Simmons and Gatesy 2015; Shen et al. 2021; Sharma and Kumar 2025). PSU offers a complementary strategy. Rather than requiring predefined partitions, it uses random subsamples of sites to assess whether support for a clade is consistent across the alignment. This does not replace model-based approaches that explicitly represent gene tree variation, but it provides a practical and computationally efficient way to detect phylogenetic conflict directly from concatenated data.

The development of *BCL* and *NBS* illustrates that PSU provides more than computational acceleration. By generating distributions of support values across independent site subsamples, PSU reveals how phylogenetic conclusions depend on different (randomly selected) subsets of the alignment and provides direct information about concordant and conflicting evolutionary signals. In this sense, PSU functions not only as a scalable computational framework but also as a diagnostic framework for evaluating the robustness of phylogenomic inference. We therefore suggest reporting *NBS* alongside *FBS* or *BCL* in analyses of concatenated phylogenomic datasets.

### Asymptotic Relationship Among FBS, BCL, and NBS

*FBS*, *BCL*, and *NBS* quantify related but distinct properties of phylogenetic support. *FBS* measures the frequency with which a clade appears in conventional bootstrap replicates generated from the full alignment. *BCL* is designed to approximate this quantity by aggregating support values obtained from many independent PSU subsamples. *NBS*, in contrast, measures the average level of support across subsamples and therefore reflects the consistency of phylogenetic signals throughout the alignment.

An important distinction is that *NBS* and *BCL* depend on the number of sites included in the subsample. When subsamples are small, different subsamples may contain different phylogenetic signals, causing *BCL* and *NBS* to be smaller than *FBS*. Even when *BCL* is the same as *FBS*, *NBS* can be substantially lower than *BCL* and *FBS* for a given subsample size. As subsample size increases, individual subsamples become increasingly representative of the full concatenated alignment and disagreement among subsamples decreases. We have found that the fraction of inferred clades supported with high bootstrap values (*f*) and the mean support (*m*) increases quickly with BCL as compared to NBS (results not shown). So, *NBS* will keep increasing with subsample size much longer than *BCL*, with *NBS* best interpreted as a scale-dependent measure of phylogenetic consistency rather than as a direct approximation of *FBS*.

In the limiting case where subsamples contain a large fraction of the alignment, PSU replicates become increasingly similar to conventional bootstrap replicates. In particular, a standard bootstrap replicate of length *N* contains only about 63.2% of the original sites at least once. Therefore, when subsamples approach this scale, PSU and conventional bootstrap analysis become the same and NBS, BCL, and FBS converge.

### Automated tuning of PSU parameters

PSU analysis requires choices for three tuning parameters: the number of sites per subsample (*n*), the number of independent subsamples (*S*), and the number of upsampling replicates per subsample (*R*). These choices affect both accuracy and computational efficiency, so adaptive protocols have been developed to select them objectively (Sharma and Kumar 2021; Sharma and Kumar 2025). The general strategy is to begin with an empirically guided subsample size and a small number of subsamples and upsampling replicates. Support values are then estimated and monitored across iterations. Additional subsamples are added until average support values (*BCL* or *NBS*) for clades in different support ranges stabilize within predefined tolerances. If convergence is not achieved, the subsample size is increased, and the process is repeated. In this case, *BCL* is used for convergence assessment across subsample sizes, rather than *NBS*, because *NBS* will keep increasing with increasing subsample size.

Once stability criteria are met, *BCL* and *NBS* are estimated from the resulting collection of PSU replicate phylogenies. This adaptive approach reduces the need for ad hoc parameter tuning and makes PSU analyses more reproducible. It is also practically important because optimal choices depend on dataset-specific properties such as alignment length, divergence, missing data, and phylogenetic complexity.

## Evolutionary Hypothesis Testing using PSU

The applications discussed thus far focused on bootstrap support estimation for phylogenetic relationships. However, the PSU framework is not limited to bootstrap support. Many core tasks in evolutionary biology involve selecting among competing models, testing evolutionary hypotheses, and comparing alternative explanations for observed sequence variation. These analyses often require repeated likelihood calculations on large alignments and can become major computational bottlenecks in phylogenomics. PSU provides a general strategy for these problems: compute the relevant likelihood-based statistic on upsampled subsamples and aggregate results across subsamples until stable conclusions are obtained. In this way, PSU extends naturally from confidence estimation to a broader class of evolutionary inference problems.

### Selecting the optimal substitution model

Choosing nucleotide or amino acid substitution models is a standard step in likelihood-based phylogenetics, typically involving likelihood-ratio tests or information criteria (Posada and Crandall 1998; Posada 2008; Kalyaanamoorthy et al. 2017; Darriba et al. 2020; Sharma and Kumar 2022). Model selection is particularly attractive for PSU because the inferential goal is discrete, the identity of the preferred model, rather than estimation of a continuous parameter, allowing convergence to be assessed directly through agreement among successive analyses.

**Figure 7.**
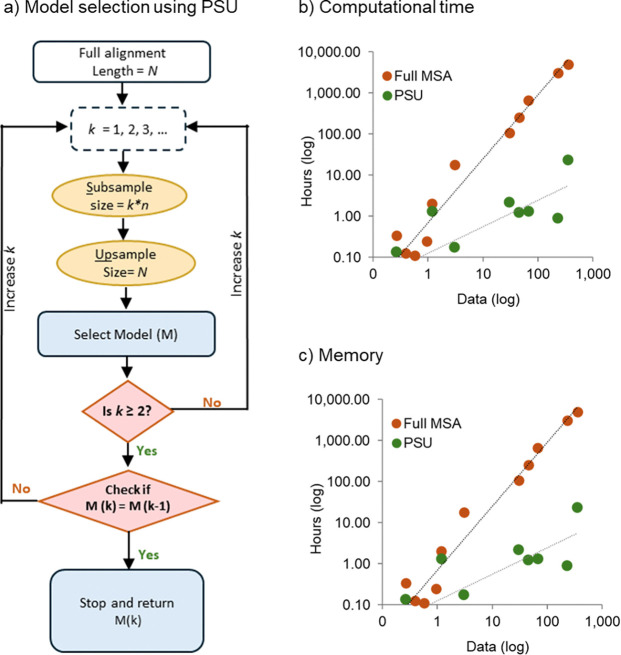
Scaling behavior of PSU versus full-data analysis for model selection. (**a**) A flowchart demonstrating the steps of the PSU framework for selecting the best-fit model of substitution. (**b**) Memory and (**c**) runtime requirements for substitution model selection as a function of data size (*D*, log scale), using data from Sharma and Kumar (2022). PSU exhibits sublinear scaling because the number of distinct site patterns evaluated remains limited, even as the total number of sites and substitutions scales to full dataset size through upsampling. PSU time and memory needs are of order *O*(*D*^0.64^) and *O*(*D*^0.23^), respectively, compared to the full-data analysis, which are of order *O*(*D*^1.56^) and *O*(*D*^1.0^), respectively, for the data set analyzed (see Figure 2).

For long phylogenomic alignments, this step can become expensive because many candidate models must be optimized and compared. PSU reduces this cost by evaluating candidate models on upsampled subsamples rather than on the full alignment (Sharma and Kumar 2022). For the 200×394 dataset, PSU selected the same final model as the full-data analysis while reducing runtime from 258 CPU hours to 1.5 CPU hours and memory use from 14 GB to 0.15 GB (Table 1). Across additional empirical datasets, PSU showed orders-of-magnitude reductions in memory use and substantially improved scaling with data size (Fig. 7b and c). For model selection, only one upsampled replicate is needed for each subsample size, since the goal is not to estimate bootstrap support. The subsample size is increased progressively until the same substitution model is selected in consecutive analyses (Fig. 7a) (Sharma and Kumar 2022). In empirical applications, convergence was usually achieved using less than 5% of the distinct site configurations in the full dataset.

### Testing nested evolutionary hypotheses

More broadly, many evolutionary questions are formulated as comparisons between nested models that differ in one or more biological assumptions. Examples include molecular clock tests, local clock models, constraints on substitution processes, and models of selective pressure. Because these analyses ultimately depend on likelihood-ratio statistics, they are natural candidates for the PSU framework. For example, the molecular clock hypothesis can be tested by comparing the log-likelihood of a rooted phylogeny under clock and no-clock models and computing the usual test statistic, 2Δ*lnL*. In a PSU implementation, the test is first conducted on an upsampled subsample. The subsample size is then progressively increased until consecutive analyses yield the same statistical conclusion at a chosen significance threshold. The final test statistic and P-value are taken from the last converged analysis. Applied to the 200×394 dataset, this protocol converged using only 4.2% of the full dataset and produced the same biological conclusion as the full-data analysis, rejecting the molecular clock with a highly significant P-value (Table 1).

Although illustrated here with the molecular clock test, the same strategy can be applied to other nested evolutionary hypotheses, including local-clock models and nested constraints on substitution processes or selection regimes, e.g., (Yoder and Yang 2000). PSU therefore provides a scalable route to likelihood-based hypothesis testing when full-data calculations may be time-consuming or impractical.

### Testing non-nested hypotheses

Non-nested comparisons represent a particularly important class of phylogenomic problems because competing hypotheses often correspond to alternative species relationships or evolutionary scenarios that cannot be compared using standard likelihood-ratio theory. In such cases, inference depends on estimating the distribution of a test statistic rather than simply selecting the model with the highest likelihood. PSU can provide an efficient framework for estimating these distributions while avoiding repeated analysis of the full alignment.

For example, PSU can be used to test tree topologies (e.g., T1 and T2). In a typical bootstrap setup, the distribution of ΔlnL = *lnL*(T1) - *lnL*(T2) can be computed across bootstrap replicates under a fixed substitution model. The null hypothesis Δ*lnL* = 0 can be tested by constructing a confidence interval for the statistic using PSU, based on the estimated variance of ΔlnL. We conducted such an analysis for two contrasting rodent trees: one from a concatenated alignment (T1) and the other from an MSC analysis (T2). They differ in the placement of a single taxon (Roycroft et al. 2020; Shen et al. 2021; Sharma and Kumar 2025).

We estimated the variance of ΔlnL across multiple upsampling replicates for each subsample, then averaged these estimates across subsamples, following the logic of Kleiner et al. (2014). Additional subsamples were added until the variance estimate stabilized within a predefined tolerance. In this example, convergence was achieved with seven subsamples. The resulting 95% confidence interval for ΔlnL ranged from −78.6 to 231.3, including 0, indicating that the concatenation tree was not significantly better supported than the alternative topology. This result is consistent with the low NBS for the defining clade of T1 and with its low posterior probability in the MSC analysis (Roycroft et al. 2020; Shen et al. 2021; Sharma and Kumar 2025).

These applications demonstrate that PSU is not restricted to confidence estimation. The same subsampling-upsampling-aggregation strategy can be used whenever the objective is to select among competing evolutionary models, test biological hypotheses, or estimate the uncertainty associated with likelihood-based statistics. This flexibility suggests that PSU should be viewed as a general computational framework for scalable evolutionary inference rather than solely as an alternative approach to bootstrap analysis.

## Estimating Evolutionary Parameters using PSU

The applications discussed thus far involve confidence estimation, model selection, and hypothesis testing. A more stringent evaluation of the PSU framework is whether it can accurately estimate continuous evolutionary parameters. Evolutionary parameter estimates are numerical quantities whose precision depends on both sampling variance and model assumptions, so the successful application of PSU to branch-length and divergence-time inference provides an important demonstration that the subsampling-upsampling-aggregation strategy can support a broad range of phylogenomic inference tasks.

### Estimating substitution model parameters

**Figure 8.**
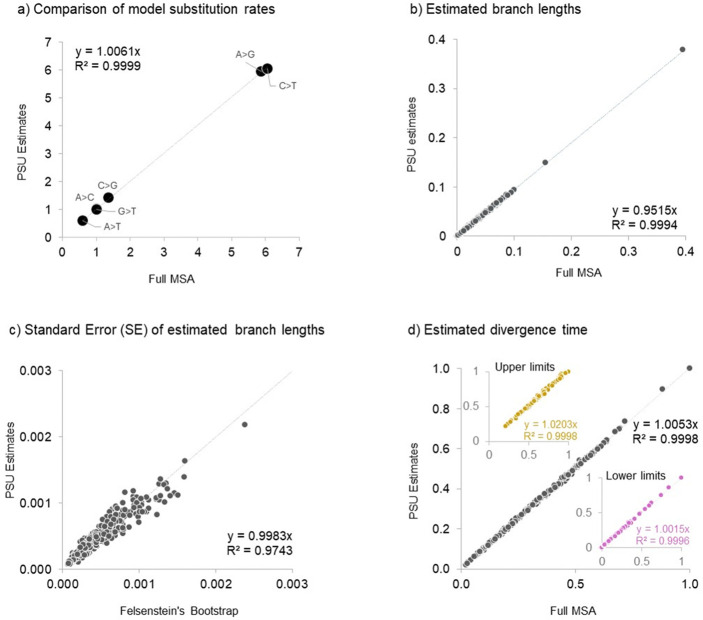
PSU estimates of model parameters, branch lengths, and divergence times for the 200×394 dataset. (**a**) Comparison of model substitution rate parameter estimates obtained from PSU and full dataset analyses. (**b**) Comparison of branch lengths for all internal and tip branches estimated using PSU and full dataset analysis. Branch lengths are estimated for the bird phylogeny and the GTR+G4 model of substitutions. (**c**) Comparison of branch length standard error (square root of estimated variance) from the standard bootstrap (x-axis) and PSU approach (y-axis). (**d**) Comparison of the relative time estimated using the RelTime approach for the given phylogeny with estimated branch lengths. The gray circle represents the relative node times, while the purple and yellow circles represent the upper and lower limits of 95% confidence intervals.

Substitution-model parameters estimated during PSU model selection were reported to be nearly identical to those obtained from full-data analysis, including the gamma shape parameter for among-site rate variation (Sharma and Kumar 2022). This suggests that PSU can be used not only to select models but also to estimate their parameters efficiently. For example, PSU can estimate the gamma shape parameter (*α*) by progressively increasing the subsample size and monitoring changes in α across iterations. The process stops when consecutive estimates fall within a predefined tolerance, 1% by default. Applied to the 200×394 dataset, this protocol converged after the fourth iteration, using at most 8.38% of all sites, and required less memory and runtime than full maximum-likelihood estimation using the complete dataset. Across empirical datasets, PSU-based estimates of *α* (0.398) closely matched full-data estimates (0.394). The same strategy can be used for substitution-rate parameters. In this case, convergence is assessed by comparing rate estimates across consecutive iterations, for example, by requiring a correlation coefficient of at least 0.99. For the 200×394 dataset, convergence was achieved after the third iteration using only 6.3% of sites, and the resulting rate estimates closely matched those from the full alignment (Fig. 8a; slope = 1.00, correlation = 0.999).

### Estimating branch lengths and their variances

Branch lengths are central to molecular evolutionary analysis because they quantify evolutionary change along lineages and are used to identify rate variation, test evolutionary hypotheses, and estimate divergence times (Tamura et al. 2012). In large phylogenomic alignments, estimating branch lengths by maximum likelihood can be computationally demanding because branch lengths and model parameters must be optimized across many site patterns. Their estimation using PSU provides a stringent test of its usefulness as a framework, as branch lengths are estimated directly from sequence variation rather than from resampling distributions.

A PSU protocol similar to that used for substitution-model parameters can be applied to branch length estimation on a given phylogeny. Here, the convergence of estimates can be assessed by monitoring the stability of short branch-length estimates as the subsample size increases. Short branches provide a stringent target because they are generally harder to estimate reliably than long branches. In our analyses, a stopping rule based on a correlation greater than 0.99 for the shortest 25% of branch lengths between successive iterations was effective.

Applied to the 200×394 dataset, this protocol produced branch-length estimates that closely matched those from the full alignment (Fig. 8b; slope = 0.95, correlation = 0.99), while reducing runtime and memory usage by 4.6-fold and 10.8-fold, respectively. PSU can also estimate branch length variances, which are needed for hypothesis testing (Dopazo 1994) and for confidence intervals in divergence-time methods such as RelTime (Tamura et al. 2012; Tamura et al. 2018; Tao et al. 2020). After a stable subsample size is identified for branch length estimation, multiple subsamples and upsampled replicates are analyzed.

Branch-specific variances are calculated within each subsample and then averaged across subsamples, following the bag-of-little-bootstraps strategy of Kleiner et al. (2014). Convergence is assessed using the coefficients of variation for the shortest 25% of branches, with estimates considered stable when the correlation between successive iterations exceeds 0.99. For the 200×394 dataset, this approach closely matched full-bootstrap variance estimates while requiring substantially less time and memory (Fig. 8c; Table 1).

### Estimating divergence times and confidence intervals.

Divergence time estimation represents one of the most computationally intensive applications in evolutionary biology because branch lengths, substitution rates, and calibration constraints must be considered simultaneously. Consequently, analyses of large phylogenomic alignments often require substantial computational resources and extended runtimes. The successful application of PSU to divergence time estimation would therefore demonstrate that the framework can accelerate not only phylogeny reconstruction but also downstream evolutionary analyses that rely on phylogenomic trees.

Indeed, divergence times obtained using the RelTime framework (Tamura et al. 2012; Tamura et al. 2018; Tao et al. 2020) using branch lengths estimated from the PSU approach were highly concordant with those estimated from the full dataset (Fig. 8d; slope = 1.005, R^2^ = 0.999). In addition to the relative node time, their 95% confidence intervals from the PSU approach closely matched those obtained from the full-dataset analysis (Fig. 8d, CI_lower_: slope = 1.001 and R^2^ = 0.999; CI_upper_: slope = 1.020, and R^2^ = 0.999).

Overall, results from this section demonstrate that PSU is not limited to estimating confidence measures or selecting among competing models. The same computational strategy can also recover continuous evolutionary parameters and their associated uncertainties. This extension from support estimation to parameter estimation substantially broadens the scope of PSU and reinforces its role as a general framework for scalable phylogenomic inference.

## Bootstrap Consensus Phylogeny and Branch Lengths using PSU

The PSU framework can also support an integrated phylogenomic workflow, in which the same upsampled replicate trees used to estimate clade support are used to construct a consensus phylogeny and produce branch lengths and substitution parameter estimates. This is useful because conventional workflows often require separate analyses for tree inference, bootstrap support, and branch length estimation. In contrast, PSU can obtain many of these quantities from a single collection of upsampled subsample analyses.

The consensus of bootstrap replicate trees is often used as the inferred phylogeny (Felsenstein 1985). Because phylogenomic datasets frequently yield strong support for most clades, PSU-derived replicate trees can like-wise be used to construct majority-rule consensus phylogenies. In such cases, clade hypotheses, support values, and estimates of phylogenetic heterogeneity are obtained in a single computational analysis.

Branch lengths and their variances can also be estimated directly from the PSU replicate trees used for building the consensus tree. For each branch in the consensus phylogeny, corresponding branch lengths are extracted from replicate trees within each subsample, and their means and variances are subsequently aggregated across subsamples. To avoid unstable estimates, variance calculations can be restricted to clades that occur in the fewest replicate trees within a subsample.

Tests using empirical datasets showed that branch-length estimates (slope = 0.95, correlation = 0.99) and branch-length variances (slope = 0.97, correlation = 0.98) closely matched those obtained from full-data analyses. These results highlight an important practical advantage of PSU: a single analysis can provide a consensus phylogeny, clade-support measures, branch-length estimates, model parameters, and associated uncertainty measures, thereby reducing the need for multiple separate full-data analyses in large phylogenomic studies.

## Applying PSU Beyond ML and Species Trees

Although this article has focused primarily on maximum-likelihood analyses of concatenated phylogenomic alignments, the PSU principle is not inherently tied to a particular inference method or taxonomic scope. More generally, PSU may be useful whenever computational cost increases with alignment size while statistical power accumulates through the addition of sites and substitutions. Under these conditions, the subsampling-upsampling-aggregation strategy can potentially reduce computational burden while preserving much of the information needed for inference.

Maximum-parsimony analyses are one potential application because computational effort increases with alignment size and repeated site patterns need not be evaluated independently. Distance-based methods, including Neighbor-Joining (Saitou and Nei 1987), may also benefit when distance estimation or bootstrap testing must be repeated many times for long alignments. Bayesian phylogenetic analyses (Holder and Lewis 2003; Yang and Rannala 2012) are particularly attractive because repeated likelihood evaluations during posterior sampling often dominate computational cost. Although the statistical behavior of PSU in Bayesian settings remains to be investigated, upsampled subsamples may provide useful approximations for estimating posterior support, branch lengths, and other evolutionary parameters.

The same logic may extend beyond species-level phylogenies. Large population-genomic datasets containing millions of polymorphic sites are increasingly used to estimate evolutionary relationships, demographic parameters, and measures of genetic diversity (Ellegren 2014; Luikart et al. 2018). Such datasets pose computational challenges similar to those encountered in species phylogenomics, suggesting that PSU-inspired approaches may prove useful in these settings as well. However, because genomic variation datasets and protein alignments often exhibit substantially greater site pattern diversity than nucleotide supermatrices, larger subsamples or modified sampling strategies may be required. Systematic evaluation of these applications remains an important direction for future research.

Thus, PSU is best viewed as a general computational template: subsample sites, upsample to full-alignment scale, compute the target quantity, and aggregate results across subsamples. Its usefulness will depend on the inference method, the computational bottleneck, and the quantity being estimated. This flexibility should allow PSU to benefit from continuing advances in scalable phylogenetic algorithms.

## Scaling with Increasing Numbers of Sequences

Until now, we have discussed how PSU addresses the computational burden in phylogenomics that arises from increasing alignment lengths. However, the number of sequences (taxa) is also growing rapidly, creating additional computational challenges (Pattengale et al. 2010; Minh et al. 2013; Sharma and Kumar 2021). Numerous methods have therefore been developed to accelerate phylogenetic inference when large numbers of taxa are analyzed, including rapid bootstrap procedures, improved tree-search algorithms, and approximate likelihood calculations (Stamatakis et al. 2008; Price et al. 2010b; Minh et al. 2013; Hoang et al. 2018). Importantly, these approaches address a different computational bottleneck than PSU. Methods such as Rapid Bootstrap and UFBoot primarily accelerate tree search and support estimation while still analyzing the full alignment. In contrast, PSU reduces the complexity of the alignment itself by limiting the number of distinct site patterns evaluated. Consequently, these approaches are complementary rather than competing and can be combined to achieve computational gains in both dimensions of a phylogenomic dataset: many sites and many taxa.

For example, Sharma and Kumar (2021) estimated subsample-wise clade support using the Ultrafast Bootstrap (UFBoot) method (Minh et al. 2013; Hoang et al. 2018). Each subsample was analyzed using UFBoot, which repeatedly upsamples the subsample alignment R times, typically R ≥ 1000, to generate replicate phylogenies from which clades and their corresponding subsample bootstrap support values, bcl, are estimated. These clade-specific bcl values are then used to estimate BCL and NBS.

For a mammalian dataset containing 37 species and 1,391,742 sites, the combined PSU+UFBoot approach required only 0.83 hours and 0.16 GB of memory on a standard multi-core computer using 10 subsamples and default UFBoot settings (Sharma and Kumar 2021). This represented a substantial improvement over using UFBoot alone, which required 7.50 hours and 6.7 GB of memory, and PSU alone, which required 18.9 hours. Similar to the FBS results, all clades except one were recovered with *BCL* ≥ 95%, whereas the remaining clade received *BCL* = 90%. These results illustrate that combining PSU with fast bootstrap heuristics can yield multiplicative gains in runtime and memory efficiency.

Other advances in phylogenetic inference can likewise be integrated into the PSU framework, including improved tree-search strategies, approximate likelihood calculations, and alternative measures of branch support such as transfer bootstrap expectation (Lemoine et al. 2018). Because PSU operates through a general subsample-upsample-compute-aggregate strategy, it can be paired with a wide range of inference engines provided that the computational burden is reduced by limiting the number of distinct sites evaluated. PSU should therefore be viewed as complementary to ongoing algorithmic advances for large phylogenomic datasets rather than as an alternative to them.

## Limitations of PSU

PSU is a theoretically motivated approximation rather than an exact reformulation of full-data phylogenetic inference. Its connection to the bag-of-little-bootstraps framework provides statistical motivation, but it does not establish equivalence between PSU and full-data analysis. At present, the strongest support for PSU comes from empirical performance across simulated and empirical phylogenomic datasets. PSU should therefore be viewed as a complementary framework that prioritizes scalability while approximating quantities that would otherwise require more expensive full-data analyses.

The effectiveness of PSU depends on whether relatively small subsamples capture representative patterns of evolutionary change. When datasets contain extensive missing data, weak phylogenetic signals, strong heterogeneity among sites, or highly uneven distributions of phylogenetic information, larger subsamples may be required to obtain stable estimates, depending on the desired evolutionary inference. In some cases, the computational advantage of PSU will diminish, and PSU will offer little benefit when the required subsample size approaches the full alignment.

For example, phylogenetic signals may be concentrated in a relatively small subset of highly informative sites. Because PSU relies on random site subsampling, some subsamples may omit these sites entirely. Although aggregation across many independent subsamples reduces this risk, datasets in which critical evolutionary information is confined to a small number of sites may require larger subsamples to reproduce conventional full-data bootstrap support values. At the same time, this behavior can be informative: when *NBS* and *FBS* differ substantially, the discrepancy may indicate that support for a clade is driven by unevenly distributed or conflicting signals (Sharma and Kumar 2025). In this sense, PSU can be useful as a diagnostic framework for situations in which information in a few sites is driving the phylogenetic signal in the analysis of concatenated alignments, e.g.(Shen et al. 2017).

Because PSU relies on random site subsampling and the number of sites subsamples, results can vary across independent runs, particularly when the number of subsamples is small or when subsample sizes are insufficient to capture representative evolutionary signals. This variability can affect estimates of clade support and other inferences. Similar run-to-run variation is common in other stochastic phylogenetic procedures, including bootstrap analyses, Bayesian posterior sampling, and heuristic tree searches. e.g., (Kumar et al. 2023). In PSU, however, such variability can be reduced by increasing the number of subsamples, increasing subsample size, or both. Adaptive protocols mitigate this problem by expanding the analysis until estimates stabilize, although optimal settings remain dataset-dependent. The computational costs reported for PSU analyses in the studies reviewed here include the adaptive tuning required to select these parameters.

Like other phylogenetic methods, PSU inherits sensitivities associated with the underlying inference procedure on concatenated sequence alignment. Model misspecification, compositional heterogeneity, long-branch attraction, or violations of other assumptions can affect PSU analyses in much the same way that they affect corresponding full-data analyses. PSU does not eliminate these challenges, although the distribution of estimates across subsamples may provide additional insight into the stability of the inferred results.

Finally, PSU does not replace methods that explicitly model particular biological processes. For example, *NBS* can reveal conflict in concatenated alignments, but it is not a substitute for multispecies coalescent models when the goal is to model gene-tree variation directly. Similarly, PSU is only useful for concatenated alignments and has not yet been systematically evaluated across all classes of evolutionary inference problems. Continued evaluation across diverse phylogenomic scenarios will be important for refining convergence criteria, identifying failure modes, quantifying computational tradeoffs, and establishing best practices for PSU analyses.

## Conclusions

PSU is a general strategy for scalable phylogenomic inference that analyzes many small representations (site subsamples) of a large concatenated alignment, upsamples them prior to inference, and aggregates the resulting estimates. Its effectiveness arises from a useful separation between computational burden and inferential power: computational cost is strongly influenced by the number of distinct site patterns in the dataset, whereas statistical power and many measures of inferential precision depend primarily on the total amount of evolutionary information represented by sites and substitutions. By reducing the former while restoring the latter through upsampling, PSU can approximate a wide range of full-data analyses at substantially lower computational cost.

The PSU framework extends well beyond accelerating bootstrap support estimation. The median of subsample support values, *BCL*, provides an accurate approximation to conventional bootstrap confidence limits, whereas the mean, NBS, incorporates conflicting subsample signals and can reduce overconfidence in concatenated phylogenomic analyses. Because PSU produces distributions of support values across independent subsamples, it also provides direct information about concordant and conflicting phylogenetic signals that is often obscured when support is summarized by a single full-data value. This makes PSU not only a computational shortcut but also a diagnostic framework for detecting heterogeneity in phylogenomic signals. The same subsampling-upsampling-aggregation strategy can be applied to substitution-model selection, likelihood-based hypothesis testing, branch-length estimation, divergence-time analysis, and variance estimation. This common computational principle unifies all current PSU applications despite their differing inferential objectives.

We have shown that a single PSU analysis can yield the consensus bootstrap phylogeny and support values, model parameters, branch lengths, and associated uncertainty measures, offering an opportunity to avoid separate full-data analyses. Adaptive procedures for selecting the subsample size, the number of subsamples, and the number of upsampling replicates further enhance the framework's practicality and reproducibility. Furthermore, methods to speed up calculations for large numbers of sequences can be used alongside PSU to improve computational efficiency for large datasets.

Although this article focused primarily on maximum-likelihood phylogenomics of species, the PSU principle is not inherently tied to a particular inference method or taxonomic scope. We anticipate that the same principle will be useful for Bayesian, maximum parsimony, and distance-based phylogenetics, as well as for large-scale population polymorphism analyses, because the computational cost increases faster than the statistical information gained from additional sites. These opportunities suggest that PSU can serve as a broadly useful computational paradigm for evolutionary analysis of massive datasets.

As phylogenomic datasets continue to grow, scalable approaches will be essential for maintaining statistical rigor and reproducibility in evolutionary inference, along with accessibility on standard computing platforms. With implementation in widely used software packages such as MEGA (Kumar et al. 2024) and growing interest in scalable phylogenomic methods, PSU is well positioned to become an important component of future big-data evolutionary analyses.

## Supplementary Material

Supplement 1

## Figures and Tables

**Table 1.** Performance of the PSU approach.

Analysis type	PSU					Full Data	
	Subsample size (max)	Savings		Time (seconds)	Memory (MBs)	Time (seconds)	Memory (MBs)
		*Time*	*Memory*				

Clade support	2.1%	79%	96%	994,142	144	4,734,008	3,773
Model selection	1.8%	99%	98%	11,160	292	928,701	14,688
Gamma parameter	8.4%	78%	91%	1,555	542	7,106	5,875
Model substitution rates	6.3%	86%	93%	980	410	7,106	5,875
Branch lengths (BLs)	8.4%	78%	91%	1,555	542	7,106	5,875
Variance (BLs)	8.4%	50%	86%	68,258	542	135,559	3,771
Molecular Clock Test	4.2%	85%	44%	2,651	1,358	17,650	2,427

**Note**. The avian phylogenomic dataset comprised 259 nuclear loci from 200 species (Prum et al. 2015). The concatenated multiple sequence alignment contains 394,686 sites and 336,490 unique site configurations.

**Figure A1.**
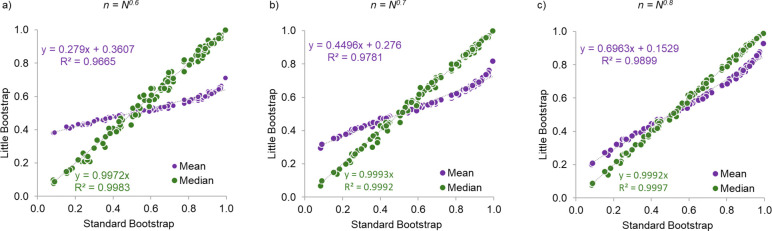
Comparison of p-values estimated by the standard and little bootstrap with mean (blue) and median bagging (orange). Data were simulated from a normal distribution X ~ *Normal*(μ = −5, σ = 1). We generated 100 random samples of size *N* = 25,000 and tested H_0_: μ = −5.01 against the one-sided alternative H_1_: μ < −5.01, a threshold chosen to produce a broad range of p-values. For each sample, p-values were estimated using the standard nonparametric bootstrap and the bag-of-little bootstraps. In the little bootstrap, subsamples of size *n* = *N*^*g*^ were drawn, with *g* = 0.6, 0.7, or 0.8 in panels **a**-**c**, respectively. For each value of *g*, 1000 subsamples were analyzed, and 100 upsampled replicates were generated from each subsample. Subsample-wise p-values were then aggregated using either the mean or the median.

## Data Availability

The phylogenomic datasets discussed in this article are publicly available from the corresponding references. R codes for performing PSU analyses are available on GitHub at https://github.com/ssharma2712.

## Appendix: Median versus Mean for Binary Scoring in Bootstrap Analysis

The little bootstrap framework requires aggregation of subsample-wise estimates. While Kleiner et al. (2014) focused primarily on mean bagging, Sharma and Kumar (2021) found that median bagging yields estimates closer to conventional bootstrap support. To illustrate why median-begging is appropriate, we considered an indicator-bootstrap framework analogous to a phylogenetic bootstrap analysis. In this setting, each bootstrap replicate receives a binary score: 1 if it supports a hypothesis and 0 otherwise. The bootstrap estimate is then the proportion of replicates that have a score of 1. This parallels phylogenetic bootstrapping, where a clade receives a score of 1 when present in a replicate tree and 0 when absent.

We generated data from a normal distribution and compared p-values obtained using the standard bootstrap with those obtained using the bag-of-little-bootstraps framework. For the little bootstrap analyses, subsamples of size (*N*^*g*^) were analyzed using both mean and median bagging. Median bagging consistently produced estimates that more closely matched those obtained from the standard bootstrap (Fig. A1). Mean bagging was more sensitive to a small number of subsamples that strongly supported or rejected the hypothesis. These extreme subsamples tend to pull the average toward 0.5, the expected value under random support. Consequently, mean bagging tends to overestimate small p-values and underestimate large p-values. A similar pattern was observed for phylogenetic bootstrap support values (Sharma and Kumar 2021), where median bagging yielded BCL values that more closely approximated conventional bootstrap support (FBS).
