# Supplementary material for: Tiny Subsamples and Upsampling Tame Big Data Evolutionary Analysis in Phylogenomics
Source: bioRxiv. 2026 Jun 22:2026.06.21.733599. Preprint. [Version 1] doi: 10.64898/2026.06.21.733599 (PMC13320971; doi:10.64898/2026.06.21.733599)
Supplement: Supplementary file 1 [file NIHPP2026.06.21.733599v1-supplement-1.pdf]

bioRxiv preprint doi: https://doi.org/10.64898/2026.06.21.733599; this version posted June 23, 2026. The copyright holder for this preprint (which was not certified by peer review) is the author/funder, who has granted bioRxiv a license to display the preprint in perpetuity. It is made available under a CC-BY 4.0 International license.

Supplementary Table S1

Data	Base type	Species	Sites	Data size (Species ✖ Sites)	Data size (Million)	DOI
Butterflies	DNA	61	5,267,461	321,315,121	321.32	https://doi.org/10.1093/sysbio/syz030
Insects A	DNA	174	3,011,544	524,008,656	524.01	https://doi.org/10.1016/j.cub.2017.01.027
Insects B	DNA	48	2,938,039	141,025,872	141.03	https://doi.org/10.1016/j.ympev.2017.12.005
Mammal A	DNA	39	1,391,742	54,277,938	54.28	https://doi.org/10.1073/pnas.1211733109
Birds	DNA	200	394,684	78,936,800	78.94	https://doi.org/10.1038/nature15697
Plants	DNA	16	4,246,454	67,943,264	67.94	https://doi.org/10.1016/j.ympev.2018.08.011
Mammal B	DNA	274	7,370	2,019,380	2.02	https://doi.org/10.1098/rspb.2012.0683
Lassa Virus	DNA	179	3,186	570,294	0.57	https://doi.org/10.1016/j.cell.2015.07.020
Vertebrate	AA	58	1,806,035	104,750,030	104.75	https://doi.org/10.1093/sysbio/syv059
Yeast	AA	23	634,530	14,594,190	14.59	https://doi.org/10.1038/nature12130
Green plants	AA	360	19,449	7,001,640	7.00	https://doi.org/10.1186/1471-2148-14-23

Supplementary Table 1*:* Data refer to genomic sequence alignments from the indicated species. Bases denote the type of nucleotide or amino acid data. Data size is calculated as the product of the number of species and the sequence length, representing the total number of aligned characters and the computational time and memory required for ML analysis. DOI provides the digital object identifiers of the studies in which these datasets were originally published and analyzed.
